# EMQN best practice guidelines for analysis and reporting of microsatellite instability in solid tumours

**DOI:** 10.1038/s41431-025-01913-x

**Published:** 2025-08-20

**Authors:** Richard Gallon, Liam McCormick, Angelica Saetta, Cristina Albuquerque, Samantha Butler, Treena Cranston, Joanne Field, Ciaron McAnulty, Patrícia Silva, Melanie Cheetham, Katie Sheils, George J. Burghel

**Affiliations:** 1 Translational and Clinical Research Institute, Faculty of Medical Sciences, Newcastle University, Newcastle upon Tyne, UK; 2 Genomic Diagnostics Laboratory, Manchester Centre for Genomic Medicine, St Mary’s Hospital, Manchester, UK; 3 1st Department of Pathology, Faculty of Medicine, National and Kapodistrian University of Athens, Athens, Greece; 4 Unidade de Investigação em Patobiologia Molecular, Instituto Português de Oncologia de Lisboa Francisco Gentil, E.P.E., Lisboa, Portugal; 5 West Midlands Regional Genetics Laboratory, Birmingham Women’s Hospital, Birmingham, UK; 6 Oxford Medical Genetics Laboratories, Oxford University Hospitals NHS Trust, Oxford, UK; 7 East Midlands Regional Molecular Genetics Service, City Hospital Campus, Nottingham University Hospitals NHS Trust, Nottingham, UK; 8 Northern Genetics Service, The Newcastle upon Tyne Hospitals NHS Foundation Trust, Newcastle upon Tyne, UK; 9 EMQN, Manchester, UK; 10 Division of Cancer Sciences, School of Medical Sciences, Faculty of Biology, Medicine and Health, The University of Manchester, Manchester, UK

**Keywords:** Diagnostic markers, Cancer genetics, Tumour biomarkers, Molecular biology

## Abstract

Microsatellite instability (MSI) is the accumulation of insertion and deletion variants (instability) in short tandem repeat DNA sequences (microsatellites). High levels of MSI occur following loss of function of the DNA mismatch repair system (MMR). MMR deficiency is an increasingly important cancer biomarker that is associated with chemotherapy resistance and response to immune checkpoint blockade, as well as one of the commonest hereditary cancer syndromes, Lynch syndrome. Since its discovery over two decades ago, our biological understanding, the testing methods, and the clinical implications of MSI analysis have expanded rapidly and up-to-date best practice guidelines are needed. An expert working group reviewed the literature and devised 15 best practice recommendations that were finalised following consultation with clinical and laboratory scientists partnered with EMQN. These include seven recommendations on key technical aspects of MSI testing and eight recommendations on the clinical interpretation and reporting of results. The latter focuses on Lynch syndrome screening and immune checkpoint blockade therapy. Example report wording is provided to assist implementation and standardisation. Common terminology and MSI analysis methods are also discussed. These guidelines are aimed primarily at genomic scientists working in diagnostic testing laboratories, but will provide a useful review of MSI for clinicians, academics, and other related professionals.

## Introduction

The DNA mismatch repair (MMR) system promotes high-fidelity DNA replication by detecting and initiating repair of base-base mismatches between parent and daughter strand. This includes single-base mismatches as well as short insertion-deletion loops. Four proteins constitute the core MMR system. MSH2 and MSH6 heterodimerise to create MutSα, which binds single-nucleotide mismatches and single-nucleotide insertion-deletion loops. MLH1 and PMS2 heterodimerise to create MutLα, which is activated by lesion-bound MutSα to nick the daughter strand and recruit additional protein complexes to excise the daughter strand and repeat synthesis. Other MMR proteins have minor roles or are components of other cellular mechanisms [1, 2].

Deficiency of MMR (dMMR) is observed across many cancer types, including colorectal and endometrial cancer, with frequencies of ~14% and 27%, respectively [3, 4]. Tumour dMMR is associated with Lynch syndrome (LS), a cancer predisposition syndrome caused by constitutional (germline) pathogenic variants affecting *MLH1*, *MSH2*, *MSH6*, or *PMS2*. Testing for dMMR in colorectal and endometrial cancers is recommended for LS screening so that identified LS patients can benefit from personalised therapy, cancer prevention, and cancer surveillance [5, 6]. dMMR tumours may not respond to certain chemotherapies [7, 8] but respond well to immune checkpoint inhibitors (ICIs) [9]. As such, dMMR is an important tumour biomarker for both LS screening and ICI therapy.

**Fig. 1:**
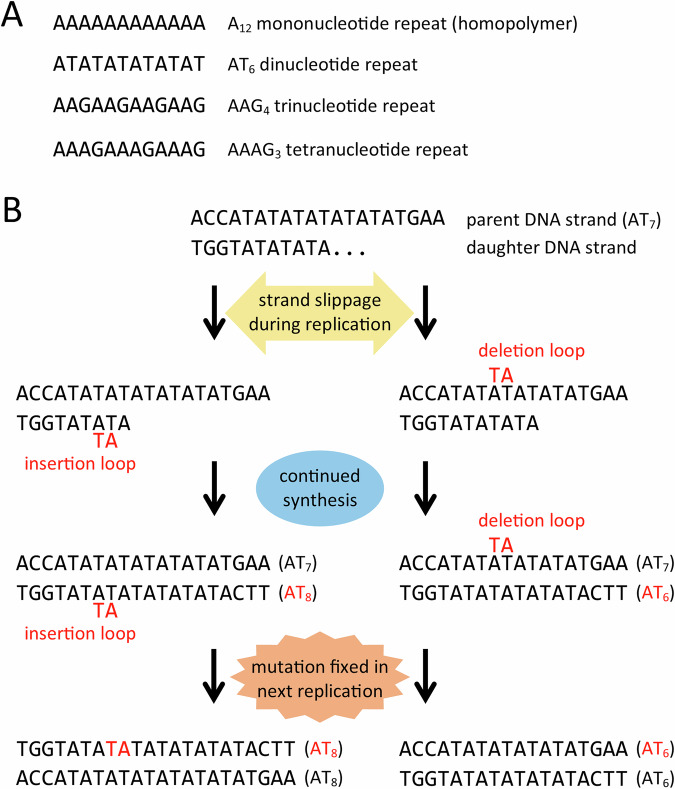
The mutational mechanism of microsatellite instability. **A** Example microsatellite sequences. **B** The strand slippage model of microsatellite insertion-deletion mutation during DNA replication.

Several tumour dMMR tests are available, including microsatellite instability (MSI) analysis. Microsatellites are tandem repeat DNA sequences where each repeat unit is composed of 1–9 (most commonly 1–6) nucleotides, referred to as mononucleotide repeats (homopolymers), dinucleotide repeats, trinucleotide repeats, and so on (Fig. 1A). Tandem repeat sequences with ten or more nucleotides in the repeat unit are called minisatellites. Microsatellites are located throughout the genome in both coding and non-coding regions [10]. Microsatellite sequence variants predominantly result from insertion and deletion mutations, with the microsatellite expanding or contracting by one or more repeat units. Several mechanisms of tandem repeat mutation have been identified, with strand slippage during DNA replication being the major contributor to microsatellite mutation [11]. Strand slippage is facilitated by the repetitive structure of a microsatellite, whereby parent and daughter DNA strands dissociate and reanneal misaligned to create either insertion or deletion loops, which become fixed variants in subsequent rounds of DNA replication (Fig. 1B). These microsatellite insertion-deletion mutations occur at a frequency several orders of magnitude higher than point mutations [10, 11]. MSI describes an increased microsatellite mutation rate and is typically quantified by the frequency of microsatellite insertion-deletion variants in a sample. Loss of MMR function leads to a ~100- to 1000-fold increase in microsatellite mutation rate [12]. Hence, high MSI (MSI-H) is a well-established biomarker of dMMR [13].

Previous guidelines for MSI analysis include the original and revised Bethesda Guidelines from meetings of the National Cancer Institute in 1996 and 2002 [13, 14]. Since then, significant advances in knowledge and technology have been made. More recent guidelines from the European Society of Medical Oncology (ESMO) and the College of American Pathologists (CAP), endorsed by the American Society of Clinical Oncology (ASCO), have provided recommendations on tumour dMMR testing to inform the use of ICIs but did not provide detailed recommendations specific to MSI analysis methodology [15–17].

The aim of these guidelines is to provide up-to-date recommendations for MSI analysis to assess solid tumour MMR status in diagnostic practice, with a focus on ICI therapy and LS risk, along with a clarification of terminology and review of common methods. These guidelines cover MSI caused by deficiency of the four core MMR proteins MLH1, MSH2, MSH6, and PMS2, whilst elevated microsatellite alterations at selected tetranucleotide repeats (which has been associated with MSH3 deficiency) is not considered due to unclear clinical relevance. These guidelines do not cover MSI analysis of cell-free DNA for cancer surveillance, monitoring, and/or prognostication and therapy decision making [18], or of non-neoplastic tissues for diagnosis of constitutional mismatch repair deficiency [19]. However, some of the following literature review and recommendations will be relevant for these settings.

## Methods

Best practice guidelines for MSI analysis methodology, interpretation, and reporting in solid tumours were deemed necessary due to the prevalent use of tumour MSI testing. To ensure widespread expert consensus, 11 representatives from relevant clinical and academic organisations across the UK and the EU were invited to share their expertise and develop guidelines. A working group was created, and the inaugural teleconference was held on the 29th September 2022. Thereafter, the team met virtually at regular intervals until 19th November 2024. The working group reviewed the literature and discussed the following points for the best practice guidelines:

1. Definition of MSI and associated terminology
2. Common MSI assays
3. MSI analysis methodology recommendations
4. MSI reporting recommendations

Group consensus was achieved on the content and order of points, with agreement to first define consistent terminology and introduce assays frequently encountered by practitioners, before providing technical recommendations relevant to the design/selection/validation of an assay and then interpretation/reporting recommendations applicable to the results of any MSI assay. Three levels of recommendation were used:

- **“must”** – essential for best practice
- **“should”** – highly advised
- **“may”** – advised where possible or practical

A guideline draft was made available through EMQN to a community of experts, including 536 clinical and laboratory scientists from institutions participating in the EMQN-organised External Quality Assessment (EQA) schemes for MSI and colorectal cancer (sporadic), and members of the Association for Clinical Genomic Science. The community consultation was held between 9th and 31st October 2024. Feedback from the consultation was appraised by the working group and the guidelines were revised accordingly.

## Results and recommendations

EMQN recommends that all laboratories offering MSI testing in solid tumours must follow established good laboratory practice (for example, Guidelines for Quality Assurance in Molecular Genetic Testing [20]). In addition, a laboratory should demonstrate compliance with internationally recognised standards (for example, ISO standards 15189: 2022 [21]), by achieving formal accreditation with a member organisation of the International Laboratory Accreditation Cooperation or equivalent national accreditation body. All tests should be validated in individual laboratories prior to implementation. EQA schemes provide further validation of testing procedures and methods; laboratories should participate annually in appropriate EQA schemes or arrange inter-laboratory exchange of samples to validate test results.

### Terminology and common MSI analysis methods

The terms used for MSI analysis vary, and errors in terminology are common. Therefore, it is important to first clarify terminology. This is further complicated by there being several common tumour MSI analysis methods, sometimes with varying terminologies used. Generally, these methods are all highly sensitive and specific assays for dMMR. Sample variables, such as tumour tissue of origin, may have a different impact on the accuracy of each method. The limit of detection of an MSI assay, i.e. the minimum dMMR tumour cell content that is detectable as an MSI-H result, varies from <1% for digital droplet PCR up to ~10% for more traditional methods. Other metrics of assay performance, such as reproducibility and robustness to sample variation, also vary, and there is a broad range of costs and turnaround times. It is outside the scope of these guidelines to provide a comprehensive review of MSI analysis methods, but the most common are introduced. More comprehensive reviews include those by Baudrin et al. [22] and Gilson et al. [23].

#### Terminology

Different terms may be used for the results of MSI analysis. “MSI-H” is used to describe a significant increase in microsatellite insertion-deletion variants that indicates dMMR. An MSI-H sample may also be described as being microsatellite unstable/instable or MSI-positive, or as having MSI. “Microsatellite stable” (MSS) refers to there being no evidence of microsatellite insertion-deletion variants and indicates proficient MMR (pMMR). Sometimes, an MSS sample is described as MSI-negative. Here, we will use MSI-H and MSS.

“Microsatellite instability-low” (MSI-L) is used to describe a moderate increase in microsatellite insertion-deletion variants detected specifically using fragment length analysis-based MSI assays. MSI-L was initially suspected of being a distinct biological category, but it was shown that, if sufficient MSI markers are analysed, most pMMR tumours contain some microsatellite insertion-deletion variants, and so MSI-L typically indicates pMMR [24–26]. However, an MSI-L result may indicate dMMR for some combinations of sample characteristics (such as tumour stage, as microsatellite insertion-deletion variants accumulate as a tumour progresses) and MSI analysis method (see Recommendations). Furthermore, next-generation sequencing-based analyses of large numbers of microsatellites have shown that the frequency of microsatellite insertion-deletion variants is a continuum between dMMR and pMMR tumours [27, 28]. Sequencing-based MSI assays may have a category of “MSI-indeterminate” (MSI-I), or an equivalent term to account for this continuum and indicate uncertainty in the result [29]. Here, MSI-L and MSI-I, though referring to different methodologies, will be used in equivalence.

Note, “dMMR” and “pMMR” refer to the underlying pathology/biology of a tumour. Therefore, whilst MSI should only be used to describe analysis of somatic insertion-deletion variants in microsatellites, dMMR/pMMR can be used to describe the result of MSI analysis or any other method used to assess MMR function, such as immunohistochemistry of MMR proteins or MMR gene sequencing. In these guidelines, descriptions of assay accuracy (such as sensitivity or specificity) refer to the ability of a method (whether DNA- or protein-based) to assess tumour MMR function.

#### Fragment length analysis

**Fig. 2:**
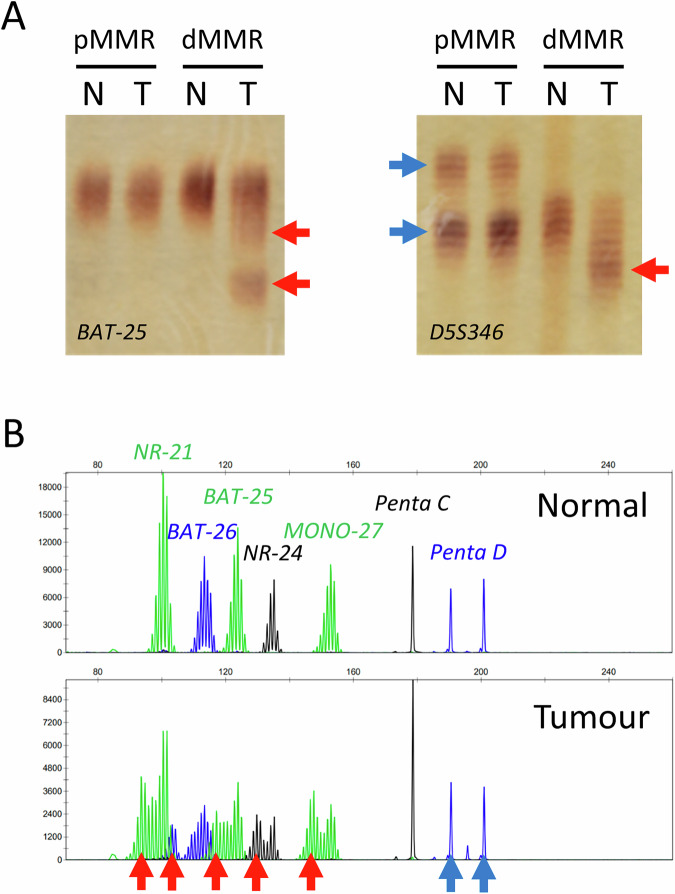
Examples of microsatellite instability analysis by fragment length analysis. **A** Example of fragment length analysis by polyacrylamide gel electrophoresis and visualisation by silver nitrate staining of amplicons for two MSI markers (BAT-25 and D5S346). Tumour (T) and matched normal (N) samples are shown from mismatch repair proficient (pMMR) and deficient (dMMR) cancers. **B** Example of fragment length analysis by capillary electrophoresis and electropherogram visualisation of fluorescently-labelled amplicons for five MSI markers (BAT-25, BAT-26, MONO-27, NR-21, and NR-24) and two polymorphic pentanucleotide repeat microsatellites for sample identification (Penta C and Penta D). Tumour and matched normal samples are shown from a dMMR cancer. The blue arrows indicate two amplicon size peaks representing heterozygous microsatellite alleles in the tumours and matched normal samples. The red arrows indicate novel amplicon sizes in the dMMR tumours compared to matched normal samples due to somatic microsatellite insertion-deletion variants.

Fragment length analysis (FLA) broadly refers to any technique by which PCR amplicons of microsatellites (MSI markers) are size-separated by electrophoresis followed by visual inspection of electropherograms to detect insertion-deletion variants (Fig. 2). FLA was the first method to detect dMMR [30, 31]. A 1996 National Cancer Institute (NCI) meeting produced the “Bethesda Guidelines” to unify LS screening using FLA and recommended specific MSI markers, including a core set of two mononucleotide repeats and three dinucleotide repeats, as well as an extended panel of 19 additional MSI markers [13]. Samples were classified by standardised thresholds of the proportion of MSI markers containing insertion-deletion variants: MSS with 0%, MSI-L with <30%, and MSI-H with ≥30% [13, 32]. A second NCI meeting in 2002 revised these recommendations and endorsed a pentaplex panel of five quasimonomorphic mononucleotide repeats [14, 33]. FLA is a well-established and widely available technique, and is considered to have a modest cost and rapid turnaround time. Limitations include the requirement for expert interpretation of stutter peak patterns and the need for matched normal DNA for interpretation of the quasi-monomorphic or polymorphic MSI markers that are often analysed [34].

#### Sequencing analysis

**Fig. 3:**
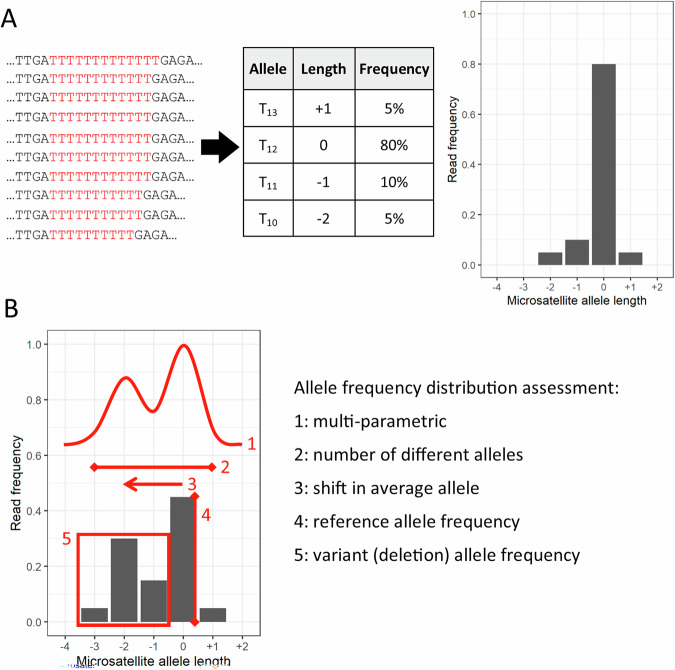
Microsatellite instability analysis by sequencing. **A** Sequencing reads containing different lengths of a microsatellite (in red) can be counted to generate a distribution of microsatellite allele frequencies, where the microsatellite allele length is given relative to the expected length from the reference genome: “0” means the microsatellite allele is the same as the reference sequence, “-1” means the microsatellite allele contains a single nucleotide deletion, “+1” means the microsatellite allele contains a single nucleotide insertion, etc. **B** Examples of the different ways in which the microsatellite allele frequency distribution can be quantified for comparison to a reference distribution from a matched normal sample or panel of microsatellite stable samples.

Many MSI assays utilising next-generation sequencing data have been developed with a variety of sequencing library preparations, bioinformatics pipelines, and classification algorithms. The number and identity of MSI markers analysed vary from a few select loci to potentially 100,000s across the genome. Sequencing-based MSI analysis typically quantifies MSI marker instability by comparison of allele distributions to reference distributions from MSS or matched normal samples (Fig. 3). MSI classification is often dichotomous (MSI-H/MSS) and thresholds vary depending on the method and MSI markers analysed. For example, a classification threshold of 20% of MSI markers showing instability has been used for gene panel and exome sequence data [27], whereas a 3% threshold has been used for genome sequence data [28]. A category of MSI-I is sometimes used to indicate samples where the MSI status cannot be clearly interpreted [29]. Some sequencing-based methods do not determine MSI status by the proportion of MSI markers showing instability and use alternative algorithms [22]. The accuracy of sequencing-based MSI analysis is dependent on robust bioinformatics pipelines tailored to the sequencing technology and panel of MSI markers used. Targeted sequencing of small MSI marker panels (10s of markers) has costs and turnaround times equivalent to FLA [35], whilst more extensive gene panel, exome, and genome sequencing have higher costs, longer turnaround times, and greater data burden [36]. Sequencing-based MSI analysis requires specialist laboratory and computational infrastructure, but these are increasingly available. Parallel analysis of other genetic markers can streamline diagnostic pathways, such as LS screening [37]. Analysis is fully automatable, facilitating high-throughput testing [36].

#### High-resolution melting curve analysis

High-resolution melting curve analysis (HRM) uses changes in the melting properties of PCR amplicons to detect microsatellite insertion-deletion variants. DNA melting is typically assessed by a double-stranded DNA-binding fluorophore. Individual MSI markers are called as stable or unstable by comparing the amplicon melting curve to a reference from an MSS sample [38, 39]. These generally analyse mononucleotide repeat MSI markers and use classification thresholds similar to FLA. HRM can immediately follow PCR in the same reaction tube, and analysis is automated [38]. HRM costs are similar to FLA, but per-sample hands-on and run times are typically <3 h. Scalability is limited by batch size.

#### Loss of MMR protein expression as an alternative dMMR biomarker

Immunohistochemistry (IHC) of tumour tissue can visualise MMR protein expression. Loss of staining of one or more proteins is a well-established, highly sensitive and specific biomarker of dMMR [13, 34, 40]. MMR IHC, therefore, provides an alternative dMMR test that features in these guidelines as it should be understood by healthcare professionals providing or interpreting results of MSI analysis. MMR IHC is widely available and has a quick turnaround time. Expert histopathologists are required for result interpretation [40]. MSI analysis and IHC assess different biological impacts of dMMR, resulting in some discordance. In particular, IHC has been shown to be insensitive in ~5% of dMMR colorectal and endometrial cancers due to retained MMR protein expression associated with MMR missense variants that disrupt MMR function but not expression [41, 42]. Conversely, dMMR cancers can lack MMR protein expression but be called MSI-L/MSS, in particular in endometrial cancers and in cancers with MSH6 deficiency [43–45]. IHC can also detect subclonal dMMR below the detection limit of common MSI analysis methods [40]. Therefore, MSI analysis and IHC should generally be considered individually adequate and complementary dMMR tests, though there may be exceptions (see Recommendation 15). More detailed considerations of the application of MMR IHC are reviewed by Chen and Frankel [46].

Note, as per WHO Guidance [47], the term “MSI” (and related terms such as “MSS”) may only be used in laboratory reports if DNA analysis has been performed and must not be used to describe the results of MMR IHC.

### Recommendations for MSI analysis methodology and validation

MSI analysis methodology refers to the entirety of the MSI assay being used, including the composition of the MSI markers, the technology used to target/amplify the MSI markers, the visualisation of microsatellite insertion-deletion variants, and the MSI classification thresholds/algorithm. These recommendations aim to provide a general basis for optimal MSI analysis methodology. These recommendations do not prescribe when MSI testing should be conducted, which should follow relevant guidelines for the specific healthcare setting. As for other oncology assays, both sample and technical quality control parameters should be established for an MSI analysis method, such as a minimum neoplastic cell content.

*Recommendation 1*: *MSI markers* ***should*** *be assessed for constitutional (germline) variants with consideration of differences between ethnic backgrounds. If using polymorphic markers, paired tumour-normal DNA* ***must*** *be analysed to exclude constitutional (germline) variants. Exceptions* ***must*** *be reported - see Recommendation 8*.

Microsatellites are some of the most variable DNA regions in the human population and are suitable for DNA fingerprinting, linkage analysis, detecting loss of heterozygosity, and similar applications where polymorphisms identify alleles—MSI was first discovered in human cancers when analysing microsatellites for these purposes [30, 31]. The suitability of polymorphic microsatellites for MSI analysis will depend on the frequency of heterozygosity, and whether tumour-normal sample pairs or methods to distinguish between constitutional (germline) and somatic variants are available. For example, quasi-monomorphic mononucleotide repeats have commonly been used for MSI analysis of tumours without matched normal samples [33], but some of these have >10% frequency of constitutional (germline) variants in African and Oceanic populations [48]. Where a significant proportion of the MSI markers being analysed are known to be polymorphic in the patient population, a matched normal sample must be analysed with the tumour to exclude constitutional (germline) variants from MSI classification [13, 14]. What defines a “significant proportion” will depend on the assay, but a guide would be to consider this significant if the presence of constitutional (germline) variants would be sufficient to (mis)classify a sample as MSI-L/MSI-I or MSI-H. Using FLA as an example, if 2/5 MSI markers are unstable and 1 of these 2 is known to be polymorphic, a matched normal DNA must be analysed as exclusion of a constitutional (germline) variant would change the MSI-H classification from instability in 2/5 markers to an MSI-L classification from instability in 1/4 markers, assuming established classification thresholds are being used [13, 32]. However, if 5/5 MSI markers are unstable and 1 of these is known to be polymorphic, a matched normal DNA does not need to be analysed as the presence of a constitutional (germline) variant would not impact classification.

*Recommendation 2*: *Only mononucleotide repeats* ***should*** *be used for MSI analysis as inclusion of longer motif microsatellites (di-, tri-, tetra-, etc, nucleotide repeats) reduces sensitivity for MSH6 deficiency. Exceptions* ***must*** *be reported - see Recommendation 8*.

MSH6 is responsible for the detection of single nucleotide mismatches and single-nucleotide insertion-deletion loops. Hence, MSH6-deficient tissues accumulate microsatellite insertion-deletion variants only in mononucleotide repeats [1, 2] and MSH6 deficiency is not detectable by MSI analysis of dinucleotide (or longer motif) repeats [49]. For the comprehensive detection of dMMR, mononucleotide repeat MSI markers should be exclusively used, and MSI results must be caveated accordingly when dinucleotide (or longer motif) repeat MSI markers are analysed—see Recommendation 8.

There is redundancy for repair of single-nucleotide insertion-deletion loops between MutSα (MSH2-MSH6) and MutSβ (MSH2-MSH3) [1, 2]. Consequently, mononucleotide repeat MSI in MSH6-deficient tissues is lower than in MLH1, MSH2, or PMS2-deficient tissues [19, 45]. Therefore, even MSI analysis of mononucleotide repeats can have decreased sensitivity for MSH6 deficiency compared to deficiency of other MMR proteins [43, 45].

*Recommendation 3*: *MSI analysis* ***must*** *use panels of multiple MSI markers, and a minimum of 5 MSI markers* ***should*** *be used to minimise the impact of constitutional (germline) variants and/or stochastic somatic mutation. Exceptions* ***must*** *be reported - see Recommendation 8*.

MSI analysis of pMMR samples, both neoplastic and non-neoplastic, will identify microsatellite insertion-deletion variants that may be constitutional (germline) variants, somatic variants that escaped MMR, or technical errors [24–26]. Within dMMR samples, stochastic mutation means that even highly sensitive MSI markers may not contain a variant. Detection of MSI may also depend on other sample-specific factors such as tumour cell divisions and clonality [50] and which MMR protein is deficient [43, 45]. Therefore, whilst individual MSI markers can achieve sensitivities and specificities >90% [33], MSI analysis requires panels of MSI markers to minimise misclassifications. Most MSI analysis methods use a minimum of five MSI markers, which is advisable to minimise the impact of constitutional (germline) variants and/or stochastic somatic mutation on classification.

*Recommendation 4*: *MSI assays* ***should*** *be clinically validated separately for each tumour type being tested, using MSI assays already validated for that specific tumour type and/or IHC as the reference method. IHC* ***should*** *be used as the reference method in non-gastrointestinal tumours. Exceptions* ***must*** *be reported - see Recommendation 8*.

The early development of MSI analysis methods focused on colorectal cancers, but the frequency and size of microsatellite insertion-deletion variants have been shown to be lower in dMMR tumours of other tissues, in particular the endometrium [25]. Furthermore, microsatellites in coding DNA sequences can be mutated at different frequencies in different tumour types due to tissue-specific selection pressures during tumorigenesis [25]. Several studies have found that commonly implemented MSI assays have lower sensitivities in endometrial than colorectal cancers [44], which is particularly notable in MSH6-deficient tumours [43, 45] and when using coding microsatellites [51]. Therefore, an MSI assay should be separately validated for each tumour type being tested.

Equivalent sensitivity and specificity of MSI analysis and IHC in colorectal and upper gastrointestinal tract cancers have been shown [16, 37, 52]. Therefore, MSI analysis as recommended by the revised Bethesda Guidelines [14] or using methods with regulatory approval (for example, CE marking) for tumour diagnostics, and/or IHC are suitable reference methods for gastrointestinal tract cancers. In non-gastrointestinal cancers, MMR IHC should be used as a reference given current evidence that suggests it may be more sensitive than MSI analysis in these tumours. Sequencing-based MSI analysis methods vary widely by technology, MSI marker panel, and classification method. Therefore, all sequencing-based MSI assays require validation against previously validated MSI analysis methods and/or IHC.

*Recommendation 5*: *Tumour mutation burden and mutational signatures* ***must not*** *be used as reference methods when validating an MSI assay*.

An increased tumour mutation burden (TMB), typically measured as the frequency of single-nucleotide and short insertion-deletion variants per megabase (Mb), has been associated with dMMR. Accurate TMB assessment requires exome or gene panel sequencing covering >1 Mb, with >10 mutations/Mb classifying tumours as having a high TMB, though this threshold varies by method [53]. However, a high TMB is not specific to dMMR tumours as there are other causes of increased mutation rate such as exogenous mutagens and polymerase proofreading deficiency. Further, TMB has relatively low sensitivity as many dMMR tumours have TMB equivalent to pMMR tumours [54]. Mutational signatures are defined by the patterns of somatic variants observed in cancers that are caused by different mutational mechanisms during tumorigenesis, and several dMMR mutational signatures have been identified [55]. Mutational signature-based classifiers of MMR status may have superior accuracy than MSI analysis or IHC [56], suggesting mutational signature analysis could become prevalent in the future. However, the need for exome or genome sequence data currently limits its clinical use and there are few/no large-scale studies demonstrating clinical efficacy. Therefore, TMB and/or mutational signatures must not be used as a reference to validate an MSI assay.

*Recommendation 6*: *Discordant results between an MSI assay and an alternative dMMR test* ***may*** *be resolved by an independent method to better characterise assay performance during validation or to assist clinical interpretation*.

Some discordance between any two dMMR tests is expected given the lack of a gold-standard method and the biological and technical differences discussed throughout these guidelines. These tests could be two different MSI analysis methods, an MSI analysis method compared to MMR protein IHC, an MSI analysis method compared to MMR gene sequencing, etc. In the case of discordance with IHC (see Recommendation 15 for reporting recommendations), the IHC staining patterns should be reviewed by a second pathologist and staining repeated if technical artefacts are suspected [40], and biological explanations should be considered (see “Loss of MMR protein expression as an alternative dMMR biomarker”). Discordant cases during clinical validation or clinical testing may be resolved using a third, independent method; for example, discordance between an MSI assay and MMR IHC may be resolved by detection of MMR (epi)genetic variants.

*Recommendation 7*: *For a novel laboratory developed test, MSI markers derived from a different MSI assay* ***should not*** *be assumed to have equivalent sensitivity and specificity: MSI markers* ***should*** *be selected for the technologies being used*.

The mutability of a microsatellite depends on several factors, including the sequence motif of its repeat unit, the number of repeat units, and its genomic context [28, 57]. Repeat motif and length are the two major determinants of microsatellite mutability in cancer [58]. If the microsatellite mutation rate is too low, an MSI marker will be stable irrespective of MMR status (low sensitivity). If too high, an MSI marker may be unstable irrespective of MMR status and/or be difficult to interpret due to technical errors (low specificity). For example, polymerase slippage during PCR amplification of MSI markers creates non-biological insertion-deletion variants that can be misinterpreted as constitutional (germline) or somatic variants [59]. The technical error rate of microsatellite insertion-deletion variant detection also depends on other aspects of the MSI analysis method. For example, FLA can be used to accurately assess relatively long microsatellites up to 60 bp in length [60], whilst next generation sequencing is limited to shorter microsatellites due to poor sequencing coverage and inaccurate read alignment of longer microsatellites, with microsatellites of 9-15 bp being the most discriminatory MSI markers in one study [58]. Microsatellite sequencing error also varies substantially between sequencing technologies [61]. Therefore, MSI marker sensitivity and specificity depend on both microsatellite mutability and analysis method. It can be assumed that the MSI markers within a commercially available MSI assay will be suitable for the methodology, but MSI markers should be carefully selected for a novel lab-developed test.

### Recommendations for reporting of MSI analysis results

These recommendations aim to aid reporting of MSI results to inform on a cancer patient’s risk of LS and responsiveness to ICI therapy given both are applicable over several types of tumour. Care should be taken to interpret results appropriately alongside other clinicopathological information. MSI status can additionally impact prognosis and response to other therapies in specific cancer types [7, 8] and should be interpreted in each clinical context. Though examples of how to present results and interpretations are given, they do not aim to be definitive as it is not possible to provide comprehensive direction for all cases and healthcare settings. Here, three possible MSI results are considered: MSS, MSI-L/MSI-I, and MSI-H (see “Terminology”). Reports should also adhere to reporting best practice, such as use of concise and lucid language, identification of the referring clinician, standardisation of patient information, and description of sample type, method, and quality control metrics.

*Recommendation 8*: *It may not be possible for a laboratory to adhere to all of Recommendations 1, 2, 3, and 4. Reports of MSI analysis results* ***must*** *include caveats specifying the limitations of the method and the implications for clinical interpretation when Recommendations 1, 2, 3, and/or 4 are not adhered to*.

Recommendation 1, 2, 3, and 4 provide guidance to minimise technical limitations of MSI analysis methods that can lead to incorrect results. Therefore, if the MSI assay used does not adhere to these Recommendations, this must be discussed within the clinical report.

For example, if polymorphic MSI markers are analysed without a matched normal DNA sample with implications for result interpretation (see Recommendation 1), a caveat must be included, such as: *“The MSI assay uses polymorphic markers. A matched normal DNA sample to exclude constitutional (germline) variants was not included in the analysis. The given result of MSI-L/MSI-I/MSI-H may not be accurate as constitutional (germline) variants may have been considered as somatic variants indicative of MSI.”*

For example, if MSI markers other than mononucleotide repeats are analysed (see Recommendation 2), a caveat must be included, such as: *“The MSI assay did not exclusively use mononucleotide repeat MSI markers. The given result of MSS/MSI-L/MSI-I may not be accurate as the di-/tri-/tetra-nucleotide repeats analysed are insensitive to MSH6 deficiency.”*

For example, if <5 MSI markers are used (see Recommendation 3), a caveat must be included, such as: *“The MSI assay used fewer than five MSI markers. The given result may not be accurate due to constitutional (germline) variants and/or stochastic somatic mutation.”*

For example, if reporting for a tumour type for which the MSI assay has not been validated (see Recommendation 4), a caveat must be included, such as: *“The MSI assay has not been validated in this tumour type and the result must be interpreted with caution.”*

*Recommendation 9*: *Reports of the MSI analysis result* ***must*** *include comment on the neoplastic cell content and any alternative histology of the sample, and the implications for clinical interpretation*.

Neoplastic cell content (NCC) must be stated on reports. If no estimate of NCC is provided with the sample for testing, this should also be stated, and results should be interpreted in the context of the validated lower limit of detection of the method used.

Where no estimate of NCC is provided, result interpretation must include a caveat, such as: “*No estimate of neoplastic cell content was provided for the pathology sample sent for analysis. The given result of MSS/MSI-L/MSI-I may not be accurate as the neoplastic cell content may be less than the validated minimum of X% for the MSI assay used.”*

Where NCC is known to be less than the validated minimum neoplastic cell content for the MSI assay used, result interpretation must include a caveat, such as: *“Neoplastic cells are reported to represent N% of the pathology sample submitted for analysis. The given result of MSS/MSI-L/MSI-I may not be accurate as the neoplastic cell content is less than the validated minimum of X% for the MSI assay used.”* Where an MSI-H (or equivalent) result is obtained, there is no need to caveat results with respect to NCC as the result indicates sufficient dMMR cells were present in the sample.

MSI analysis, as well as MMR IHC, can be affected by alternative cell content or tissue structure, for example, necrosis and abundant mucinous components as well as low neoplastic cellularity [62, 63]. Therefore, reports of MSI analysis results for samples with any exceptional histology must be caveated, such as: “*Assuming the dysplastic cells in the sample tested are representative of neoplastic cells, these results indicate…*”

In cases with inadequate NCC, it would be appropriate to request a new sample with enriched NCC or to explore alternative dMMR tests, such as MMR IHC or a different MSI assay, and this must be included in the report, for example: *“The neoplastic cell content of the sample was insufficient to provide a reliable result due to* [specific reason from pathology] *and a new sample/alternative MSI assay/mismatch repair immunohistochemistry has been requested.”*

In benign tumours/dysplastic lesions, there are currently no clinical recommendations for dMMR testing for LS screening or to inform therapy. Whilst MSI testing of a benign tumour/dysplastic lesion may be informative for LS risk, for example approximately 70% of LS colorectal adenomas have dMMR, it is far less definitive than testing of a cancer [4]. Where a sample has insufficient NCC or alternative histology, MSI testing of a different tumour sample should be requested if available, especially where family history, patient age, or other such clinical indicators suggest the patient may have LS.

*Recommendation 10*: *Reports of an MSS/MSI-H result for colorectal cancers and endometrial cancers* ***must*** *include a statement on the likelihood of a Lynch syndrome diagnosis*.

The tumours of LS patients are characterised by dMMR due to somatic (epi)mutation of the second allele of the constitutionally- (germline-) affected MMR gene. Guidelines from international expert groups recommend dMMR testing of colorectal and endometrial cancer patients to identify patients at-risk of LS who should be offered constitutional (germline) genetic testing of MMR genes [5, 6]. These guidelines are supported by health economic analyses that show LS screening in colorectal and endometrial cancer patients is a cost-effective intervention due to the benefits of cancer risk management available to LS carriers once identified [64, 65]. Given this international consensus, any report of MSI analysis of colorectal or endometrial cancers must state the likelihood of an LS diagnosis based on the result.

A report of an MSS result should include the following clinical interpretation or similar: “*The MSS result is consistent with proficient DNA mismatch repair and reduces the likelihood of a diagnosis of Lynch syndrome*.” A report of an MSI-H result should include the following clinical interpretation or similar: “*The MSI-H result is consistent with deficient DNA mismatch repair and increases the likelihood of a diagnosis of Lynch syndrome*.” For how to report MSI-L/MSI-I results with respect to the likelihood of an LS diagnosis, please see Recommendation 14.

*Recommendation 11*: *Activation or the results of additional tests of MSI-H colonic and endometrial cancers to support interpretation of the likelihood of Lynch syndrome* ***may*** *be included in the report*.

Additional tumour tests are recommended following an MSI-H result to exclude sporadic dMMR tumours from constitutional (germline) genetic testing for LS [5, 6]. In dMMR colonic cancers, this includes either testing for NM_004333.6(*BRAF*):c.1799T>A p.(Val600Glu) or *MLH1* promoter hypermethylation, where detection of either *BRAF* c.1799T>A p.(Val600Glu) or *MLH1* promoter hypermethylation indicates the tumour is likely sporadic in origin (typically via the sessile serrated pathway of colonic tumorigenesis) and that the likelihood of LS is reduced. As these biomarkers are largely redundant, one or both tests are acceptable for the exclusion of sporadic dMMR colonic cancers from LS screening [66]. Note, these additional tumour tests are only relevant for colonic cancers and not rectal cancers. The sporadic pathway of dMMR tumorigenesis, associated with the *BRAF* c.1799T>A p.(Val600Glu) variant and *MLH1* promoter hypermethylation, does not generally occur in rectal cancer. Therefore, dMMR rectal cancers have a high probability of LS and should trigger constitutional (germline) genetic testing without additional tumour tests [3].

In dMMR endometrial cancer, the follow-on tests do not include testing for *BRAF* c.1799T>A p.(Val600Glu) as this variant is rare and has no implication for an LS diagnosis, but do include testing for *MLH1* promoter hypermethylation, which is indicative of a sporadic tumour and reduced likelihood of an LS diagnosis [67].

Note, if results of MMR IHC are also available, *BRAF* c.1799T>A p.(Val600Glu) variant and/or *MLH1* promoter hypermethylation testing are only needed when the tumour shows loss of MLH1 expression (see Recommendation 15).

Reports of an MSI-H result for colonic and endometrial cancers may include activation of subsequent tumour tests. For example, a report of an MSI-H colonic cancer may include the following or similar: “*The MSI-H result is consistent with deficient DNA mismatch repair. Testing for BRAF c.1799T*>*A p.(Val600Glu) and/or MLH1 promoter hypermethylation has been activated to determine the likelihood of a diagnosis of Lynch syndrome and will be reported separately*.”

If available, reports may include the results of *BRAF* c.1799T>A p.(Val600Glu) and/or *MLH1* promoter hypermethylation testing along with an MSI-H result. *BRAF* status, for instance, may be available with MSI status following tumour sequencing [37]. For example, when *BRAF* c.1799T>A p.(Val600Glu) and/or *MLH1* promoter hypermethylation have not been detected, the clinical interpretation may read as follows or similar: “*The MSI-H result is consistent with deficient DNA mismatch repair. BRAF c.1799T*>*A p.(Val600Glu)/MLH1 promoter hypermethylation was not detected in the tumour. The combination of MSI-H and absence of BRAF c.1799T*>*A p.(Val600Glu)/MLH1 promoter hypermethylation increases the likelihood of a diagnosis of Lynch syndrome*.” For example, when *BRAF* c.1799T>A p.(Val600Glu) and/or *MLH1* promoter hypermethylation have been detected, the clinical interpretation may read as follows or similar: “*The MSI-H result is consistent with deficient DNA mismatch repair. BRAF c.1799T*>*A p.(Val600Glu)/MLH1 promoter hypermethylation was detected in the tumour. The combination of MSI-H and presence of BRAF c.1799T*>*A p.(Val600Glu)/MLH1 promoter hypermethylation increases the likelihood of a sporadic origin and reduces the likelihood of a diagnosis of Lynch syndrome*.”

Note, there are limitations to the current recommendations for LS screening using molecular testing of tumours and so the results of MSI analysis and these additional tumour tests should be considered alongside other characteristics of the patient (such as age of cancer diagnosis, family history of cancer, etc) that may also indicate constitutional (germline) genetic testing for LS [4]. In particular, whilst there is an approximate 80% concordance between *MLH1* promoter methylation testing and *BRAF* c.1799T>A p.(Val600Glu) testing, *MLH1* promoter hypermethylation identifies more sporadic dMMR tumours and so is the more specific biomarker within the LS screening pipeline [68]. However, approximately 2-3% of *MLH1*-associated LS patients have constitutional *MLH1* promoter hypermethylation, and ~15% of *MLH1*-associated LS colorectal cancers have promoter hypermethylation as the second hit in *MLH1*, hence *MLH1* promotor hypermethylation testing may exclude LS patients from screening [4]. Furthermore, constitutional (germline) genetic testing may not identify an MMR pathogenic variant despite the patient having a dMMR colonic or endometrial cancer without *BRAF* c.1799T>A p.(Val600Glu) variant and/or *MLH1* promoter hypermethylation. The majority of these LS-negative cases have double somatic MMR pathogenic variants [37, 69] and some guidelines recommend tumour MMR gene sequencing to confirm the tumour is sporadic in origin and so reduce diagnostic uncertainty [70].

*Recommendation 12*: *Reports of an MSS/MSI-H result for other (non-colorectal, non-endometrial) cancers* ***may*** *include a statement on the likelihood of a Lynch syndrome diagnosis, considering relevant guidelines for the specific healthcare setting*.

dMMR is found across many types of cancer and is associated with LS irrespective of the tissue of origin [4, 29]. dMMR testing of other (non-colorectal, non-endometrial) cancers within the LS spectrum (including gastric, hepatobiliary, pancreatic, small bowel, ovarian, prostate, urothelial, sebaceous, and glial tumours) could be a useful LS screening strategy [4], but clinical guidelines recommending this are currently lacking. Whilst MSI analysis of other tumour types is not routine in many healthcare settings, it may be done to inform the use of ICI therapy (see Recommendation 13) and is likely to become increasingly common as part of a cancer patient’s diagnostic work up. Therefore, in any cancer, irrespective of tissue of origin, an MSI-H result indicates the patient is at increased risk of LS and this may be included in the report following relevant guidelines for the specific healthcare setting. Similar wording to that suggested in Recommendation 10 could be used. Note, the value of additional tumour tests to exclude sporadic dMMR cases in other cancer types may not be clear, and should follow guidelines for the specific healthcare setting.

*Recommendation 13*: *Reports of an MSS/MSI-H result for any cancer* ***must*** *include a statement on the likelihood of response to immune checkpoint inhibitor therapy, considering relevant guidelines for the specific healthcare setting*.

Growing evidence supports a common response to ICI therapy by dMMR tumours across tissue types whether sporadic or associated with LS [9]. ICIs are also the first therapy to be given a tissue-agnostic approval by the FDA as a second-line treatment in any MSI-H metastatic solid tumour [71]. The ESMO guidelines for dMMR testing to inform use of ICI therapy are focused on tumours of the LS-spectrum, in which dMMR is more common [15], whilst the CAP/ASCO guidelines are less prescriptive with respect to tumour type [16, 17].

A report of an MSS result must include the following interpretation, or similar: *“The MSS result is consistent with proficient DNA mismatch repair and reduces the likelihood of response to immune checkpoint inhibitor therapy.”* A report of an MSI-H result must include the following interpretation, or similar: *“The MSI-H result is consistent with deficient DNA mismatch repair and increases the likelihood of response to immune checkpoint inhibitor therapy.”*

Note, other biomarkers, such as TMB, may also indicate the likelihood of response to ICIs irrespective of MSI status and should be considered along with results of MSI analysis if available.

*Recommendation 14*: *An MSI-L/MSI-I result* ***should not*** *be interpreted as evidence of proficiency or deficiency of MMR and* ***must not*** *be interpreted to indicate response to immune checkpoint inhibitor therapy, though* ***may*** *be interpreted to increase the likelihood of a Lynch syndrome diagnosis*.

MMR status cannot be clearly interpreted from an MSI-L/MSI-I result due to a continuum in MSI between dMMR and pMMR tumours, though MSI-L/MSI-I is mostly associated with pMMR (see “Terminology”), and should be reported using the following or similar: “*The MSI-L/MSI-I result does not clearly predict DNA mismatch repair proficiency/deficiency*.”

An alternative dMMR test, such as MMR IHC (see Recommendation 15), should be used to clarify the MSI-L/MSI-I result.

Due to the broad adverse event profile of ICIs [72], no comment should be made on the likelihood of response to ICI therapy.

MSI-L/MSI-I may be associated with LS, especially in endometrial and other non-colorectal cancers [29]. Furthermore, the potential harms to the patient of reporting an MSI-L/MSI-I result as increasing the likelihood of LS are much less significant than using an MSI-L/MSI-I result to determine use of ICI therapy. Therefore, a report of an MSI-L/MSI-I result may include comment on the likelihood of an LS diagnosis, with consideration of local guidelines on LS screening and the frequency of MSI-L/MSI-I results to ensure the burden of additional downstream testing is manageable. For example, a report may state: *“The MSI-L/MSI-I result does not clearly predict DNA mismatch repair proficiency/deficiency but increases the likelihood of a diagnosis of Lynch syndrome*.” The activation or results of further testing to exclude sporadic dMMR cases from LS screening may be included in the report along with the MSI-L/MSI-I result as for an MSI-H result (see Recommendation 11).

*Recommendation 15*: *If relevant and available, results of MMR IHC* ***may*** *be included in the report and used to aid interpretation of the MSI analysis result*.

MMR IHC is a complementary test to MSI analysis (see “Loss of MMR protein expression as an alternative dMMR biomarker”). MMR IHC results are particularly useful when the MSI analysis method has limitations (see Recommendation 8) or produces an MSI-L/MSI-I result (see Recommendation 14), or when the sample has an NCC below the detection limits of the MSI analysis method (see Recommendation 9). Therefore, if relevant and readily available, results of MMR IHC may be used in the interpretation of the MSI analysis result. If results are concordant, a simple statement can be made, such as: *“Immunohistochemistry of mismatch repair proteins found* [state result with respect to all four MMR proteins] *and the tumour to be deficient/proficient in DNA mismatch repair, consistent with the MSI-H/MSS result.”* If the results are discordant, careful interpretation is needed, and a third dMMR test should be considered to resolve discordance (see Recommendation 6). An exhaustive discussion of the possible causes of discordance is beyond the scope of this recommendation (see “Loss of MMR protein expression as an alternative dMMR biomarker”). Non-canonical MMR staining patterns and correlation with MSI status have previously been discussed [73].

When MSI-H or MSS and discordance with IHC cannot be resolved, the result should be reported to indicate dMMR, in agreement with CAP guidelines for dMMR testing of tumours for ICI therapy [16, 17]. When MSI-L/MSI-I, the result of MMR IHC should be used to interpret whether the tumour is dMMR or pMMR.

The results of MMR IHC can also determine the optimal LS screening pathway and, if available, may be included in the interpretation of the likelihood of an LS diagnosis (see Recommendations 10-12). In particular, sporadic dMMR colonic and endometrial cancers are most frequently caused by loss of *MLH1* expression [66, 67]. Therefore, if MMR IHC is available, *BRAF* c.1799T>A p.(Val600Glu) and/or *MLH1* promoter hypermethylation test activation or results should only be included in the report of an MSI-H colonic or endometrial cancer that shows loss of MLH1 and PMS2 expression. Loss of expression of MSH2 and MSH6, MSH6 in isolation, or PMS2 in isolation, increases the likelihood of an LS diagnosis without need for *BRAF* c.1799T>A p.(Val600Glu) and/or *MLH1* promoter hypermethylation testing. Note, *BRAF* c.1799T>A p.(Val600Glu) testing of a dMMR endometrial cancer is not informative (see Recommendation 11).

### Recommendations summary

**Fig. 4:**
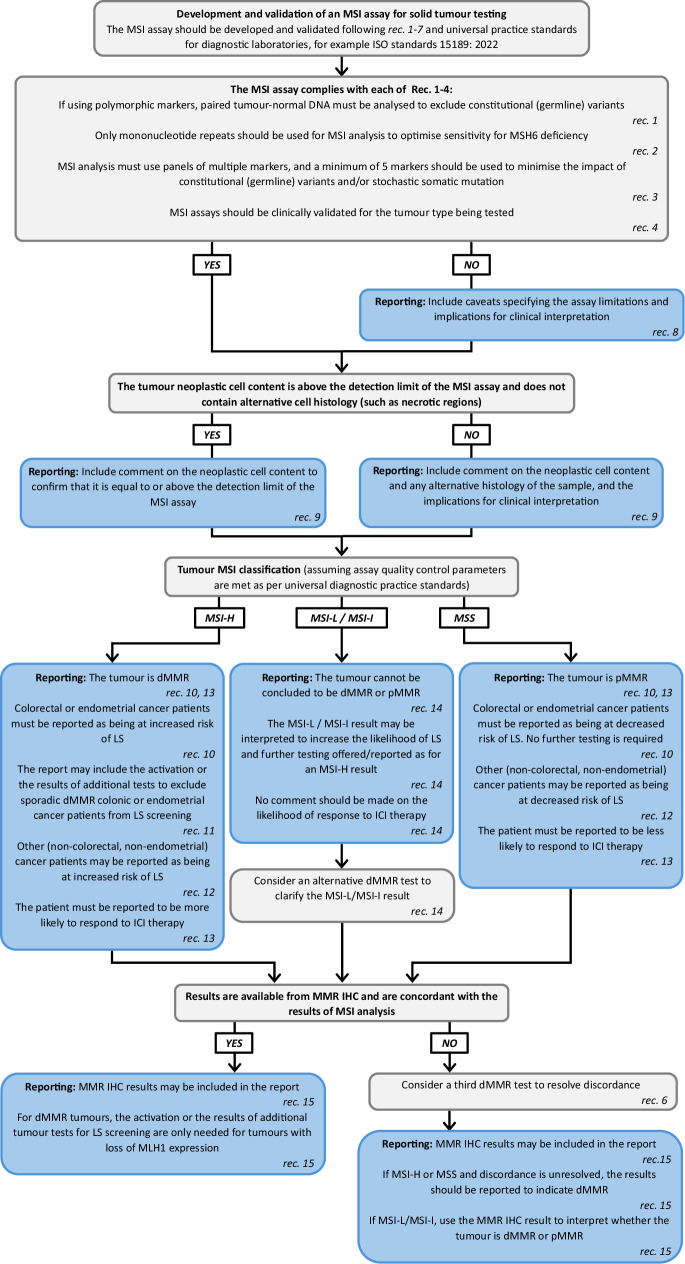
Diagnostic and reporting workflow for microsatellite instability analysis. Steps for diagnostic practice and reporting of microsatellite instability (MSI) analysis of solid tumours are presented along with summaries of best practice recommendations (rec.). For ISO standards 15189:2022, please see [21]. Please refer to the main guidelines text for additional details. dMMR mismatch repair deficient, ICI immune checkpoint inhibitor, IHC immunohistochemistry, LS Lynch syndrome, MMR mismatch repair, MSI-H microsatellite instability-high, MSI-L/MSI-I microsatellite instability-low / microsatellite instability-indeterminate, MSS microsatellite stable, pMMR mismatch repair proficient.

Recommendations are summarised in Table 1 and a diagnostic and reporting workflow is provided in Fig. 4.

**Table 1:** Recommendations summary.

**Recommendations for MSI analysis methodology and validation**	
Recommendation 1	MSI markers **should** be assessed for constitutional (germline) variants with consideration of differences between ethnic backgrounds. If using polymorphic markers, paired tumour-normal DNA **must** be analysed to exclude constitutional (germline) variants. Exceptions **must** be reported - see Recommendation 8.^1^
Recommendation 2	Only mononucleotide repeats **should** be used for MSI analysis as inclusion of longer motif microsatellites (di-, tri-, tetra-, etc, nucleotide repeats) reduces sensitivity for MSH6 deficiency. Exceptions **must** be reported - see Recommendation 8.
Recommendation 3	MSI analysis **must** use panels of multiple MSI markers, and a minimum of 5 MSI markers **should** be used to minimise the impact of constitutional (germline) variants and/or stochastic somatic mutation. Exceptions **must** be reported - see Recommendation 8.
Recommendation 4	MSI assays **should** be clinically validated separately for each tumour type being tested, using MSI assays already validated for that specific tumour type and/or IHC as the reference method. IHC **should** be used as the reference method in non-gastrointestinal tumours. Exceptions **must** be reported - see Recommendation 8.^2^
Recommendation 5	Tumour mutation burden and mutational signatures **must not** be used as reference methods when validating an MSI assay.
Recommendation 6	Discordant results between an MSI assay and an alternative dMMR test **may** be resolved by an independent method to better characterise assay performance during validation or to assist clinical interpretation.^3^
Recommendation 7	For a novel laboratory developed test, MSI markers derived from a different MSI assay **should not** be assumed to have equivalent sensitivity and specificity: MSI markers **should** be selected for the technologies being used.^4^
**Recommendations for reporting of MSI analysis results**	
Recommendation 8	It may not be possible for a laboratory to adhere to all of Recommendations 1, 2, 3, and 4. Reports of MSI analysis results **must** include caveats specifying the limitations of the method and the implications for clinical interpretation when Recommendations 1, 2, 3, and/or 4 are not adhered to.
Recommendation 9	Reports of the MSI analysis result **must** include comment on the neoplastic cell content and any alternative histology of the sample, and the implications for clinical interpretation.
Recommendation 10	Reports of an MSS/MSI-H result for colorectal cancers and endometrial cancers **must** include a statement on the likelihood of a Lynch syndrome diagnosis.
Recommendation 11	Activation or the results of additional tests of MSI-H colonic and endometrial cancers to support interpretation of the likelihood of Lynch syndrome **may** be included in the report.^5^
Recommendation 12	Reports of an MSS/MSI-H result for other (non-colorectal, non-endometrial) cancers **may** include a statement on the likelihood of a Lynch syndrome diagnosis, considering relevant guidelines for the specific healthcare setting.
Recommendation 13	Reports of an MSS/MSI-H result for any cancer **must** include a statement on the likelihood of response to immune checkpoint inhibitor therapy, considering relevant guidelines for the specific healthcare setting.
Recommendation 14	An MSI-L/MSI-I result **should not** be interpreted as evidence of proficiency or deficiency of MMR and **must not** be interpreted to indicate response to immune checkpoint inhibitor therapy, though **may** be interpreted to increase the likelihood of a Lynch syndrome diagnosis.
Recommendation 15	If relevant and available, results of MMR IHC **may** be included in the report and used to aid interpretation of the MSI analysis result.

Please refer to the main guidelines text for additional details. IHC immunohistochemistry, MMR mismatch repair, MSI-H microsatellite instability-high, MSI-L/MSI-I microsatellite instability-low/microsatellite instability-indeterminate, MSS microsatellite stable.

^1^For example, if 2/5 MSI markers are unstable and 1 of these 2 is known to be polymorphic, a matched normal DNA must be analysed as a constitutional (germline) variant would change the MSI-H classification from instability in 2/5 markers to an MSI-L classification from instability in 1/5 markers, assuming established classification thresholds are being used [13]. However, if 5/5 MSI markers are unstable and 1 of these is known to be polymorphic, a matched normal DNA does not need to be analysed as the presence of a constitutional (germline) variant would not impact classification.

^2^MSI analysis as recommended by the revised Bethesda Guidelines [14] or using methods with regulatory approval for tumour diagnostics are suitable reference methods for gastrointestinal tract cancers.

^3^Examples of an independent method include gene panel sequencing to detect MMR variants or testing for *MLH1* promoter hypermethylation.

^4^It can be assumed that the MSI markers within a commercially available MSI assay will be suitable for the methodology.

^5^Examples of additional tests include testing for *BRAF* p.Val600Glu and/or *MLH1* promoter hypermethylation.

## Discussion

These guidelines provide an overview of common terminology and methods for MSI analysis, and 15 recommendations for best practice. The recommendations aim to mitigate the major technical limitations of different MSI analysis methods. In particular, the use of panels of mononucleotide repeat MSI markers is recommended to enhance MSI detection in MSH6-deficient tumours, in agreement with the recent ESMO guidelines [15]. An MSI analysis method should be validated against MMR IHC in non-gastrointestinal tumours, in accord with the recent CAP/ASCO guidelines [16, 17]. Reporting guidelines focus on two clinical associations with dMMR cancers: ICI therapy response and risk of LS. LS screening recommendations focus on colorectal and endometrial cancers due to the wealth of evidence in these tumour types [5, 6]. However, whilst there is no best practice consensus currently, LS risk may be reported in other tumour types [4, 29]. Healthcare practitioners should also be aware that dMMR may have other clinical implications in specific tumour types that are not discussed in these guidelines [7, 8].
